# Supplementary material for: Content-rich biological network constructed by mining PubMed abstracts
Source: BMC Bioinformatics. 2004 Oct 8;5:147. doi: 10.1186/1471-2105-5-147 (PMC528731; doi:10.1186/1471-2105-5-147)
Supplement: Additional File 2 — The original results of the above study (non-essential files are deleted to keep the file size under the limit set by BMC bioinformatics). [file 1471-2105-5-147-S2.bz2 › chilibotAdditionalFile2/dip10/36ID7539137E131/html/FKBP1A_FRAP1.html]

 


 **FKBP1A** and **FRAP1** 
  
Found 77 abstracts in PubMed, retrieved 10.  
 

 What does Google say? 
 PDF only 
| .edu only 

---

**Interactive relationship** (e.g. stimulation, inhibition, etc)

**Inhibitory relationship**- Rapamycin FKBP12  [ **FKBP1A** ]  inhibited mTOR  [ **FRAP1** ]  activity.  Ref: 12370290 Mol Cell Biol, 2002
- In essence, these agents gain function by binding to the immunophilin FK506 binding protein 12  [ **FKBP1A** ]  and the resultant complex inhibits the activity of mTOR  [ **FRAP1** ] .  Ref: 12864941 Clin Breast Cancer, 2003
**Neutral relationship**

**Non-interactive relationship** (e.g. studied together, co-existance, homology, etc.)

- The FKBP12  [ **FKBP1A** ]  rapamycin associated protein FRAP  [ **FRAP1** ]  is a CLIP 170 kinase.  Ref: 12231510 EMBO Rep, 2002
- In complex with its cellular receptor, the FK506 binding protein FKBP12  [ **FKBP1A** ] , rapamycin binds and inhibits the function of the mammalian target of rapamycin  [ **FRAP1** ]  mTOR  [ **FRAP1** ] .  Ref: 12234610 Biochem Pharmacol, 2002
- In this study, we show that FKBP12  [ **FKBP1A** ]  rapamycin associated protein FRAP  [ **FRAP1** ] , also called mTOR  [ **FRAP1** ]  RAFT  [ **FRAP1** ]  interacts with CLIP 170.  Ref: 12231510 EMBO Rep, 2002
- Upon BMP4 treatment, the serine threonine kinase FKBP12  [ **FKBP1A** ]  rapamycin associated protein FRAP  [ **FRAP1** ] , mammalian target of rapamycin  [ **FRAP1** ]  mTOR  [ **FRAP1** ] , associates with Stat3 and facilitates STAT activation.  Ref: 12796477 J Cell Biol, 2003
- Rapamycin inhibits the FK506 binding protein 12  [ **FKBP1A** ]  mammalian target of rapamycin  [ **FRAP1** ]  complex and causes cell cycle arrest in G1.  Ref: 12599018 Urol Res, 2003
- Such activation of p70 S6K is blocked by pharmacological inhibitors of the PI 3 kinase or the FKBP 12  [ **FKBP1A** ]  rapamycin associated protein mammalian target of rapamycin  [ **FRAP1** ]  FRAP  [ **FRAP1** ]  mTOR  [ **FRAP1** ] .  Ref: 12759354 J Biol Chem< MedlineTA>J Biol Chem, 2003
- The signal from DNA damage to activation of these response pathways appears to be mediated by FKBP12  [ **FKBP1A** ]  rapamycin associated protein FRAP  [ **FRAP1** ] , since these phosphorylation events are blocked by rapamycin.  Ref: 12231188 Methods, 2002
- Rapamycins first bind a cyclophilin FKBP12  [ **FKBP1A** ] , and this complex binds and inhibits the function of mTOR  [ **FRAP1** ]  mammalian target of rapamycin  [ **FRAP1** ]  a serine threonine Ser Thr kinase with homology to phosphatidylinositol 3 kinase.  Ref: 12878853 Cancer Biol TherCancer Biol Ther, 2002
- eIF4G Delta and Delta eIF4G were fused C and N terminally to the FK506 binding protein FKBP  [ **FKBP1A** ]  and the FKBP  [ **FKBP1A** ]  rapamycin binding domain FRB of the human FKBP  [ **FKBP1A** ]  rapamycin associated protein FRAP  [ **FRAP1** ] , respectively.  Ref: 12768627 Biotechnol Bioeng, 2003
